# A Prospective Comparative Study of Laparoscopic Common Bile Duct Exploration and Endoscopic Retrograde Cholangiopancreatography for Managing Common Bile Duct Calculi

**DOI:** 10.7759/cureus.82827

**Published:** 2025-04-23

**Authors:** Ranendra Hajong, Bijit Medhi, Pinky Rabha, Arup J Baruah, Polina Boruah, Sunny Aggarwal, Khumanthem M Devi, Lomtu Ronrang, Samapti Debnath

**Affiliations:** 1 Surgery, North Eastern Indira Gandhi Regional Institute of Health and Medical Sciences (NEIGRIHMS), Shillong, IND; 2 General Surgery, North Eastern Indira Gandhi Regional Institute of Health and Medical Sciences (NEIGRIHMS), Shillong, IND; 3 Biochemistry, North Eastern Indira Gandhi Regional Institute of Health and Medical Sciences (NEIGRIHMS), Shillong, IND; 4 Anesthesiology, North Eastern Indira Gandhi Regional Institute of Health and Medical Sciences (NEIGRIHMS), Shillong, IND; 5 Transfusion Medicine, North Eastern Indira Gandhi Regional Institute of Health and Medical Sciences (NEIGRIHMS), Shillong, IND; 6 Dentistry, North Eastern Indira Gandhi Regional Institute of Health and Medical Sciences (NEIGRIHMS), Shillong, IND

**Keywords:** calculi, common bile duct, endoscopic retrograde cholangiopancreatography, laparoscopic common bile duct exploration, stone clearance

## Abstract

Introduction: Common bile duct (CBD) calculi are typically managed by either laparoscopic CBD exploration (LCBDE) or endoscopic retrograde cholangiopancreatography (ERCP). The superiority of one method over the other remains a matter of debate. This study was designed to compare the efficacy and morbidity profile of LCBDE and ERCP in the management of CBD calculi.

Materials and methods: This analytical cross-sectional study was conducted from January 2021 to July 2024. A total of 72 patients with CBD calculi were included, with 36 undergoing LCBDE and 36 undergoing preoperative ERCP followed by laparoscopic cholecystectomy. The primary endpoint was the complete stone clearance rate from the CBD via LCBDE (using either a trans-cystic or supra-duodenal approach) or at the first ERCP procedure. Morbidity and mortality outcomes were also assessed.

Results: The stone clearance rate was significantly higher in the LCBDE group (88.89%) compared to the ERCP group (72.22%), with an OR of 3.077 and a 95% CI of 0.864-10.954. Retained CBD calculi were noted in 10 patients in the ERCP group, compared to four in the LCBDE group. Two patients in the ERCP group developed acute pancreatitis with elevated amylase and lipase levels. Biliary leaks were observed in two patients in the LCBDE group and one in the ERCP group who succumbed to multidrug-resistant sepsis.

Conclusion: LCBDE demonstrates higher efficacy in clearing CBD calculi compared to ERCP, especially in patients with multiple and large CBD calculi, with a lower morbidity profile.

## Introduction

Stones in the common bile duct (CBD) most commonly result from the passage of gallstones through the cystic duct into the CBD [1,2]. Less frequently, they may originate in the CBD itself. More than 10-18% of patients undergoing cholecystectomy for gallstones also have concomitant CBD stones [3].

Laparoscopic CBD exploration (LCBDE) and endoscopic retrograde cholangiopancreatography (ERCP) are effective modalities in the management of CBD calculi. LCBDE was found to be superior to ERCP in achieving stone clearance in some series (OR: 2.89; 95% CI: 1.81-4.61) [4,5]. Failure rates with conventional ERCP are higher for removing large stones and can reach up to 20%, warranting additional procedures [6].

LCBDE is a single-stage approach with reduced complications but requires expertise in advanced surgical skills [7]. ERCP, on the other hand, is a two-stage procedure and may fail to clear multiple stones or large impacted stones in the CBD with a higher risk of pancreatitis [8].

This study was designed to compare the stone clearance rate of LCBDE and ERCP at first and to assess the morbidity and mortality profile of both procedures.

## Materials and methods

Study design

This analytical type of cross-sectional study was conducted from January 2021 to December 2024 at a tertiary care hospital in North East India.

Inclusion and exclusion criteria

Patients with a confirmed diagnosis of CBD calculi, as determined by abdominal ultrasonography or magnetic resonance cholangiopancreatography, were included in the study after obtaining informed consent. Patients who did not consent and those with concomitant malignancies were excluded from the study. The primary objective of the study was to compare the efficacy of complete stone clearance by LCBDE with the first ERCP in managing CBD calculi. The secondary objectives included mortality and morbidity profiles, such as the incidence of retained stones and post-procedure complications like pancreatitis and biliary leak.

Data collection

The sample size was calculated based on an estimated prevalence of CBD calculi of 5%, with a power of 80%. The Institutional Ethics Committee of North Eastern Indira Gandhi Regional Institute of Health and Medical Sciences (NEIGRIHMS) issued approval NEIGR/IEC/M12/F12/2020 on September 18, 2020. A total of 72 patients with CBD calculi were included in the study after obtaining informed consent. Thirty-six patients underwent LCBDE along with cholecystectomy in the same sitting, while the remaining 36 patients underwent ERCP followed by laparoscopic cholecystectomy in two stages. LCBDE was performed either through a trans-cystic or supra-duodenal choledochotomy using a flexible choledochoscope or a rigid nephroscope, and compared with the stone clearance rate at the first ERCP procedure. Patients were randomly allocated to the LCBDE or ERCP group using computer-generated random numbers. Data from patients were collected on a pre-designed pro forma and entered into Microsoft Excel (Microsoft Corp., Redmond, WA, USA) periodically.

Statistics analysis

Statistical analyses of the collected data were performed using MedCalc statistical software (MedCalc Software Ltd., Ostend, Belgium), which is available online. Continuous variables are presented in percentages. Comparison of the proportions between the two groups was performed using the chi-square test, and a two-by-two table was used to calculate the OD between the groups. A p-value of 0.05 or less was considered statistically significant.

## Results

A total of 72 patients participated in the study: 15 were male and 57 were female. The clinical profiles of both groups were comparable in terms of age, BMI, CBD diameter, and the number of CBD calculi, among other factors, as shown in Table 1.

**Table 1:** Clinico-radiological profile of the LCBDE and ERCP groups LCBDE: laparoscopic common bile duct exploration, ERCP: endoscopic retrograde cholangiopancreatography, CBD: common bile duct, BMI: body mass index, CI: confidence interval, SE: standard error

Variables (n=72)	LCBDE (n=36)	ERCP (n=36)	p-value	t-statistic/chi-square	95% CI	SE
Males (15)	7	8	0.77	0.083	-16.013 to 21.367	NA
Females (57)	29	28	0.597			
Age (years)	39.27 ± 11.2	38.73 ± 10.9	0.84	-0.207	-5.735 to 4.655	2.605
BMI (kg/m2)	23.2 ± 3.44	22.9 ± 2.56	0.68	-0.420	-1.725 to 1.125	0.715
CBD diameter (cm)	1.22 ± 0.27	1.16 ± 0.31	0.38	-0.892	-0.194 to 0.074	0.067
Number of CBD calculi	4.72 ± 3.11	4.48 ± 2.99	0.74	-0.334	-1.674 to 1.194	0.719
Stone diameter (cm)	1.75 ± 0.92	1.62 ± 0.87	0.54	-0.616	-0.551 to 0.291	0.211

**Figure 1:**
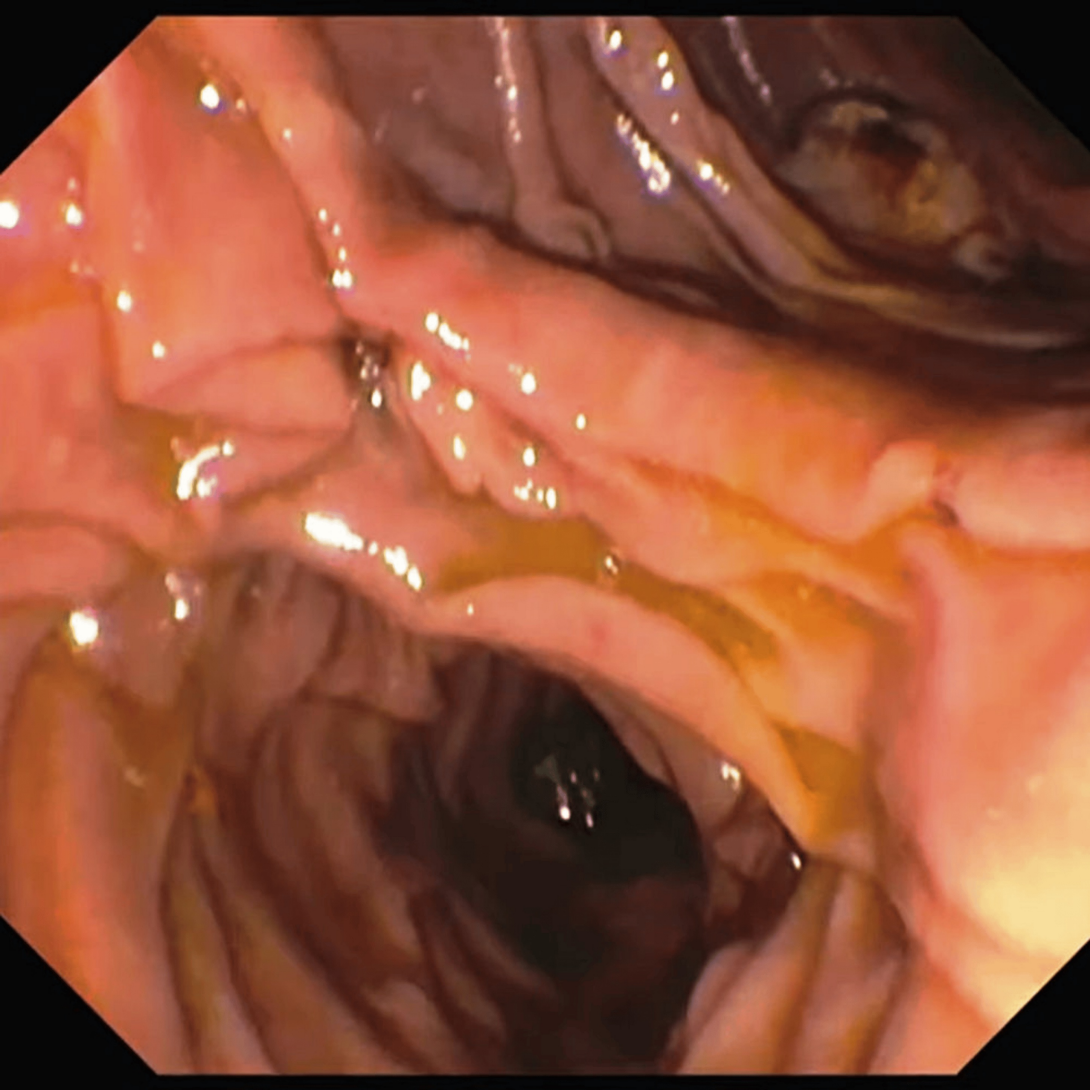
Extracted stone in the duodenum using ERCP ERCP: endoscopic retrograde cholangiopancreatography

The complete stone clearance rate was higher in the LCBDE group compared to patients undergoing their first ERCP procedure (n=32; 88.89% vs. n=26; 72.22%), with an OR of 3.077 and a 95% CI of 0.864 to 10.954. Six patients in the ERCP group who had retained stones underwent repeat ERCP, as shown in Figure 1, while the remaining four patients underwent LCBDE. In the LCBDE group, two patients with retained stones underwent ERCP, and two underwent repeat LCBDE.

Two patients developed acute pancreatitis following ERCP and were managed conservatively. Two patients in the LCBDE group developed minor biliary leaks. Radiologically guided tube drainage was performed in both patients, followed by ERCP. In the ERCP group, one patient developed a biliary leak following duodenal wall perforation and ultimately succumbed to uncontrolled sepsis, as shown in Table 2.

**Table 2:** Post-procedure outcome measures LCBDE: laparoscopic common bile duct exploration, ERCP: endoscopic retrograde cholangiopancreatography, OR: odds ratio, CI: confidence interval

Parameters (n=72)	LCBDE (n=36)	ERCP (n=36)	p-value	OR	95% CI	Z statistic
Complete stone clearance (%)	32 (88.89)	26 (72.22)	0.08	3.077	0.864 to 10.954	1.735
Retained stones	4	10	0.08	0.325	0.091 to 1.157	1.735
Pancreatitis	0	2	0.27	0.178	0.008 to 3.847	1.101
Biliary leak	2	1	0.58	2.059	0.178 to 23.774	0.579
Perforation	0	1	0.49	0.324	0.012 to 8.227	0.683

**Figure 2:**
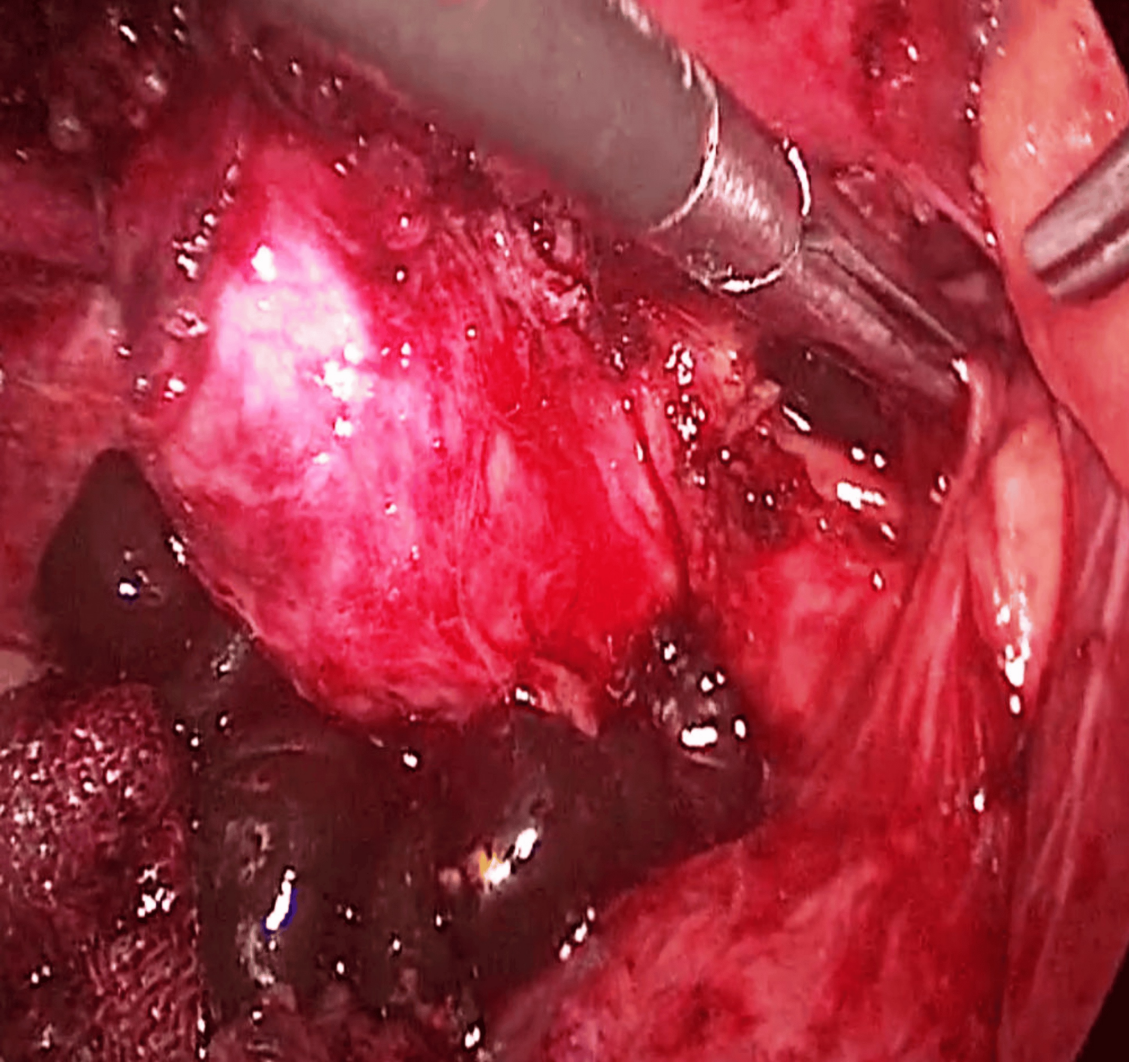
Multiple CBD calculi CBD: common bile duct

Trans-cystic LCBDE was performed in seven patients, and supra-duodenal choledochotomy was performed in the remaining 29 patients, as shown in Figure 2.

Retained stones were found in two patients undergoing trans-cystic LCBDE and were subsequently managed by supra-duodenal choledochotomy using LCBDE. Small missed stones were detected in two patients undergoing supra-duodenal choledochotomy in the LCBDE group, who were subsequently managed by ERCP.

## Discussion

Single-stage LCBDE with cholecystectomy or two-stage ERCP followed by cholecystectomy are the standard management options available for CBD calculi, depending on local expertise and equipment availability. However, there is no consensus on the superiority of one technique over the other, and management of CBD stones continues to present a dilemma [9-13].

A major disadvantage of ERCP is that it is a two-stage procedure and is associated with life-threatening complications such as pancreatitis, bleeding, and duodenal perforation. It has also been reported that sphincterotomy may cause papillary stenosis and an increased risk of bile duct cancer. The inability to retrieve large stones is also a major limitation [14].

In the present study, the overall clearance rate of CBD stones was lower in both the LCBDE and ERCP groups, which may be attributed to the large number of stones and the presence of large and impacted stones. However, the overall rate of stone clearance was higher in the LCBDE group, with an OR of 3.077, although the difference was not statistically significant (p=0.08).

Liu et al. in their series also found a higher efficacy rate for LCBDE than for ERCP (93.3% vs. 82.5%) in clearing CBD calculi, which was statistically significant (p<0.05), with a similar morbidity and mortality profile [15]. Zhang et al. demonstrated a statistically significant (p < 0.05) higher overall efficacy rate for LCBDE compared to ERCP (97.1% vs. 76.6%) in their study on managing CBD calculi after cholecystectomy [16].

The overall complication rates were higher in the ERCP group in the present study, similar to the series by de Silva et al. [17]. Zhang et al. found LCBDE to be superior to ERCP, with a better stone clearance rate in terms of stone diameter and quantity and lower hospital costs [16]. Similar results were found in the present study, with a higher and larger number of calculi detected in the CBD.

However, ERCP remains the predominant management strategy worldwide, with many published series reporting excellent outcomes [18]. Two patients in the LCBDE group developed minor biliary leaks (5.56%), compared to one patient in the ERCP group (2.78%). Wu et al. also reported the incidence of biliary leaks after LCBDE and ERCP as 7.5% and 2.5%, respectively [19]. Acute pancreatitis post-ERCP was reported in two patients (5.56%), which is consistent with the previously reported series [20]. However, Wu et al. reported a very high incidence of acute pancreatitis post-ERCP (20%) [19]. Death can occur in less than 1% of cases post-ERCP due to acute pancreatitis, perforation, or cardiorespiratory complications [21-23]. One patient succumbed to sepsis post-ERCP perforation in the present series (2.78%).

## Conclusions

ERCP is used universally for removing CBD calculi. However, LCBDE scores better in patients with multiple and larger CBD calculi with fewer postoperative complications and reduced need for additional interventions. ERCP is not the ideal treatment modality for managing multiple and large CBD calculi. However, for small stones, both procedures are equally effective, and the choice of procedure depends on the availability of equipment and expertise.
